# Occurrence of measles in a country with elimination status: Amplifying measles infection in hospitalized children due to imported virus

**DOI:** 10.1371/journal.pone.0188957

**Published:** 2018-02-15

**Authors:** HyeEun Eom, YoungJoon Park, JooWhee Kim, Jeong-Sun Yang, HaeJi Kang, Kisoon Kim, Byung Chul Chun, Ok Park, Jeong Ik Hong

**Affiliations:** 1 Division of Vaccine-Preventable Diseases Control and National Immunization Program, Korea Center for Disease Control and Prevention, Cheongju-si, Chungcheongbuk-do, Republic of Korea; 2 Division of Respiratory Viruses, Korea National Institute of Health, Cheongju-si, Chungcheongbuk-do, Republic of Korea; 3 Department of Preventive Medicine, Korea University Medical College, Seongbuk-gu, Seoul, Republic of Korea; Public Health England, UNITED KINGDOM

## Abstract

The Republic of Korea declared measles elimination in 2006. However, a measles outbreak occurred in 2013. This study aimed to identify the epidemiological characteristics of the sources of infection and the pattern of measles transmission in 2013 in South Korea. We utilized surveillance data, epidemiological data, immunization registry data, and genetic information. We describe the epidemiological characteristics of all measles case patients (sex, age distribution, vaccination status, sources of infection) as well as details of the outbreak (the pattern of transmission, duration, mean age of patients, and generation time). In 2013, a total of 107 measles cases were notified. Most patients were infants (43.0%) and unvaccinated individuals (60.7%). We identified 4 imported and 103 import-related cases. A total of 105 cases were related to four outbreaks that occurred in Gyeongnam, northern Gyeonggi, southern Gyeonggi, and Seoul. The predominant circulating genotype was B3 type, which was identified in the Gyeongnam, northern Gyeonggi, and southern Gyeonggi outbreaks. The B3 type had not been in circulation in South Korea in the previous 3 years; virologic evidence suggests that these outbreaks were import-related. Most measles cases in South Korea have been associated with imported measles virus. Although Korea has maintained a high level of herd immunity, clustering of susceptible people can cause such measles outbreaks.

## Introduction

The incidence of measles declined markedly worldwide after introduction of the measles vaccine. Countries aiming to eliminate measles have launched catch-up, keep-up, and follow-up immunization campaigns against measles infection and some have achieved measles elimination [1]. Measles was eliminated in the Americas in the year 2000 and in 2014 among four countries in the Western Pacific region [2, 3]. In 1965, South Korea first introduced one-dose measles-containing vaccine (MCV), given at 9 months of age; in 1983, trivalent measles–mumps–rubella (MMR) vaccine was included in the National Immunization Program (NIP), given at age 9–15 months. In 1997, a two-dose MMR vaccine schedule was implemented, with the first dose given at age 12–15 months and the second at age 4–6 years. South Korea changed from a disease control to an elimination policy after 2001, when a massive measles outbreak occurred that affected about 5.5 million people, even after the introduction of MCV [4, 5]. After implementing a 5-year plan, which included mandatory epidemiologic investigation, a school entry requirement for two doses of MMR vaccination, and active laboratory surveillance, measles incidence has decreased to less than one case per million. Indeed, South Korea has achieved almost all interim target of measles elimination, as defined in 2004 by the World Health Organization Regional Office for the Western Pacific (WHO/WPRO). This plan has resulted in the elimination of endemic measles virus transmission in South Korea [6].

In countries that have implemented successful strategies, such as including two-dose MCV in the NIP, well-functioning measles surveillance systems, and virus isolation in most cases, endemic measles has been eliminated, as certificated by the WHO. However, some countries have experienced a change in the epidemiology and clinical characteristics of the disease [7–9]. For example, the United States (US) achieved measles elimination in 2000; however, there has been a change in the age distribution of patients in the country [9]. Indeed, a low incidence of measles reduces the opportunity for physicians to see patients with suspected measles; this situation may hamper the early detection of measles. Missed opportunities for early detection and inadequate measles preventative measures are closely related to the undetected transmission of a disease and occurrence of a disease outbreak. Having specific information on the changed epidemiology and nonspecific clinical manifestation in a low-incidence situation is crucial. However, the epidemiology of measles has not been studied since 2011 in South Korea [10–12]. Epidemiological information also forms the basis of preparation for a future measles outbreak, and such studies should be conducted in a collaborative manner between government and primary medical and health institutions [13]. This study aimed to provide needed information on the most recent dynamics of measles by year, in a country that has declared elimination of indigenous measles transmission.

## Materials and methods

### Data sources

This study utilized data from four sources: measles surveillance data, epidemiological survey data, immunization records from the Immunization Registry System, and genetic information from analyses of measles virus.

As part of the measles surveillance system in South Korea, based on the Infectious Disease Prevention Act, measles has been a mandatory reportable disease since 2001. All suspected cases of measles must be reported to the National Notifiable Disease Surveillance System (NNDSS) of the Korea Centers for Disease Control and Prevention (KCDC). Epidemiological Intelligence Service (EIS) officers are placed in each province to monitor infectious disease. Upon notification, an EIS officer performs an epidemiological investigation and reports the findings to the KCDC. In this study, we confirmed all reported cases of measles to the NNDSS during the period January to December 2013.

Epidemiological investigation data included general information about the reported case patient (sex, age, disease onset, diagnosis date, laboratory test results) as well as additional information (rash onset, measles-associated symptoms, complications, international travel history, domestic activities (business, meeting, travel) including response activities (isolation, contact tracing, post-exposure prophylaxis), affiliation, and mortality).

We also used data from the immunization history to identify vaccination status, utilizing a computerized immunization registry system (IR, the national immunization registry), which has been operated by the KCDC since 2003. The viral genotype was determined from throat swab samples by the Korea National Institute of Health (KNIH) in Chungcheongbuk-do, South Korea.

All Korean measles surveillance data are available at https://is.cdc.go.kr/dstat/index.jsp. All statistical data of notifiable infectious diseases in Korea, including measles, can be accessed and queried by anyone. This website is maintained in Korean language only. Epidemiologic survey data and personal immunization records can be accessed by authorized staff of the KCDC (contact: evenlyehs@korea.kr). Korean law provides for the protection of personal information under the Personal Information Protection Act (PIPA), in effect from September 2011. Therefore, all epidemiological survey data and personal identification information are encrypted and stored securely in a government database.

### Case definition and classification

The WHO/WPRO definition of measles cases and infection sources was applied in this study [14]. A suspected measles case was defined as any person with an acute fever; maculopapular rash, and one or more of the following symptoms: cough, coryza, or conjunctivitis. A laboratory-confirmed measles case was defined as any person with measles-compatible symptoms and one or more positive laboratory results (i.e., positive measles-specific IgM, 4-fold increase in IgG titer in paired serum samples, a positive result based on RT-PCR or virus isolation). An epidemiologically confirmed case was defined as a case without laboratory confirmation but that was geographically and temporally related to a laboratory-confirmed case [14].

To determine the sources of infection, we classified cases as imported, import-related, endemic, and unknown, according to WHO guidelines. Imported cases were defined as those that were exposed to measles abroad during the 7–21 days prior to rash onset. Import-related cases were defined as those directly linked to imported cases or that were related to imported virus. Endemic cases were defined as those infected by endemic measles virus that had been in circulation during the previous 12 months in a specific area of South Korea. Unknown cases were defined as those that were impossible to classify.

The location of infection was defined as the place where case patients were contacted to a previous measles case. In this study, we classified the location of infection into four categories: hospital, school (including kindergarten), household, and community.

A measles outbreak was defined as a case with a clear epidemiological relation to one or more cases. Generation time, which is useful for monitoring and tracking measles, was defined as the time interval between the day on which rash onset occurred in the first case and the day on which rash onset occurred in a secondary case [15].

### Laboratory methods

Laboratory confirmation was defined as a positive result for one or more of the following: measles-specific IgM, 4-fold increase in IgG titer in paired serum samples, RT-PCR, or virus isolation. All samples were processed at the KNIH, which was designated the National Measles Laboratory (NML) by the WHO in 2006. Measles-specific antibodies were identified in serum samples using a measles ELISA test for IgM and IgG (Siemens Healthcare Diagnostics Inc., Erlangen, Germany). Throat swab samples were frozen until shipment for RT-PCR testing and virus isolation. Measles genotypes were identified using the RT-PCR-mediated target gene (N gene) directly from clinical specimens or from measles virus isolates, followed by sequencing. All measles nucleotide data in Korea are reported to the WHO Measles Nucleotide Surveillance system (http://who-measles.org/Public/Web_Front/epi.php).

### Epidemiological assessment

This descriptive study analyzed the epidemiological characteristics of measles cases in 2013. We used descriptive statistics, obtained with IBM SPSS version 20 (IBM Corp., Armonk, NY, USA). General characteristics of the case patients were described (sex, age distribution, and vaccination status) as well as outbreak details (transmission patterns, duration, mean age of patients, and generation time). Furthermore, we classified all cases according to the source of infection. Mean generation time was estimated only in cases that had been definitely linked to another case epidemiologically.

## Results and discussion

### Descriptive epidemiology of measles cases

**Fig 1:**
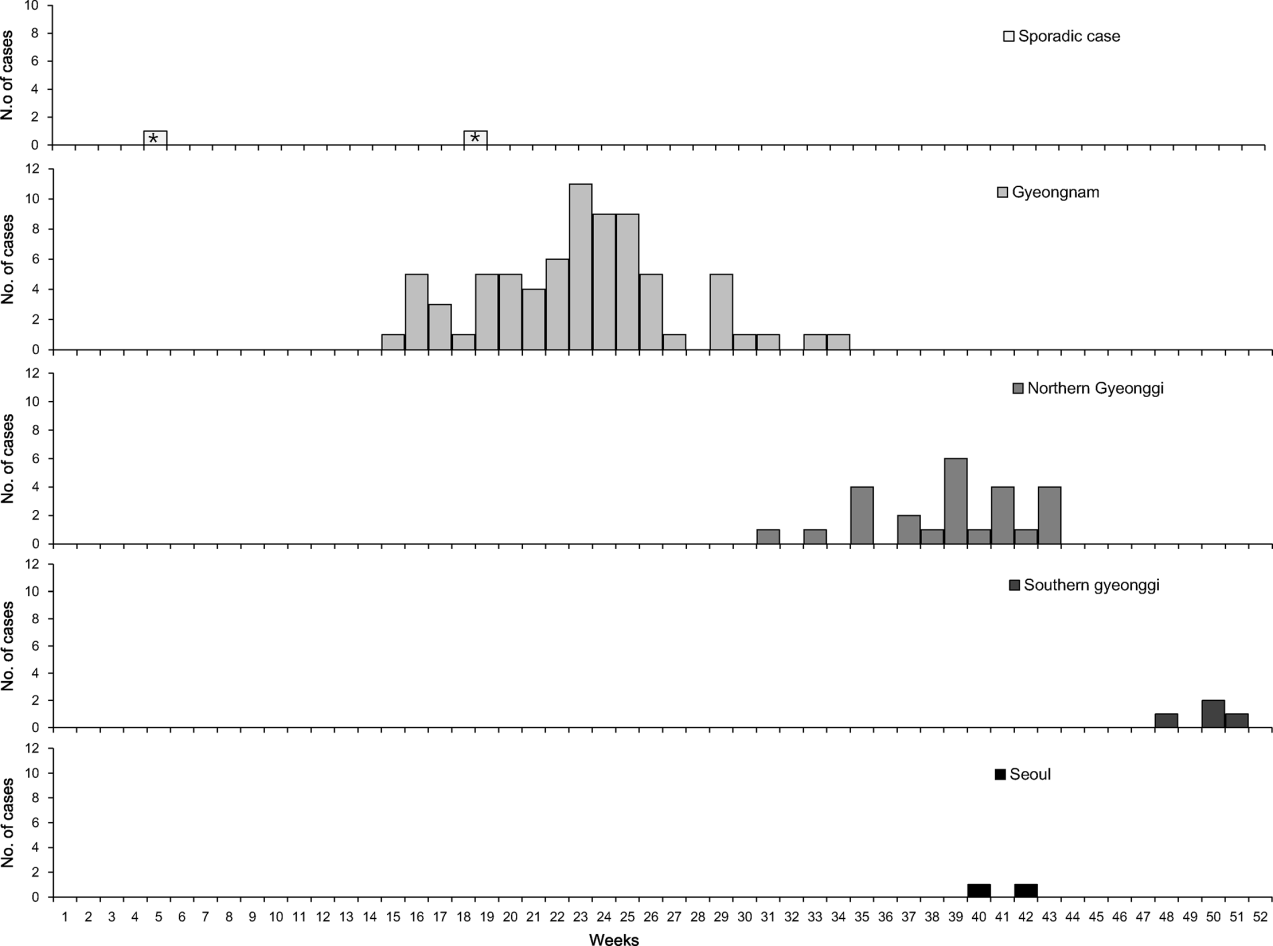
Sporadic cases and four outbreaks by week, according to sources of infection, in South Korea, 2013. *Reference: date of rash onset *Sporadic cases detected in weeks 5 and 18 lived in Gyeongnam and Southern Gyeonggi provinces, respectively. No sporadic cases were related to the four outbreaks.

**Fig 2:**
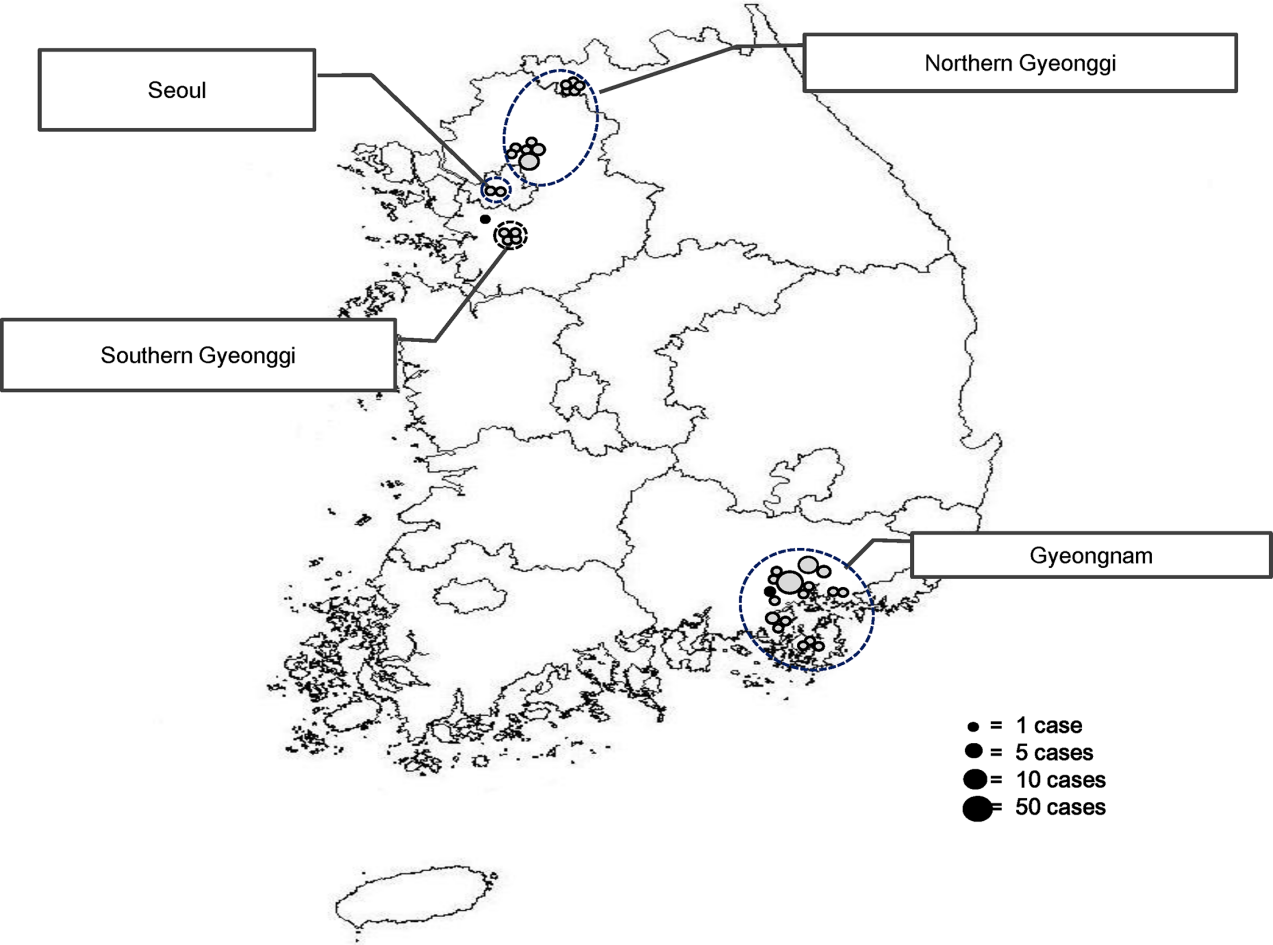
Geographical distribution of measles cases in South Korea, 2013. *A foreigner (n = 2) is displayed in notification area. Black dot on the map indicates a sporadic case. Measles outbreaks occurred in four regions of South Korea (Gyeongnam, northern and southern Gyeonggi, and Seoul).

In 2013, a total of 107 measles cases were reported in South Korea, of which 92.5% occurred in Gyeongsangnam-do (Gyeongnam) and Gyeonggi-do (Gyeonggi) provinces (Figs 1 and 2). Of the total measles case patients, 50.5% (n = 54) were male (Table 1). The predominantly affected age group was infants <1 year (43.0%; n = 46); 18.7% (n = 20) of patients were aged 20 years or older, 17.8% (n = 19) were aged 1–3 years, and 14.0% (n = 15) were aged 13–19 years. Among the 92 case patients with known vaccination status, only 4 had received two doses of MMR vaccine. A total of 65 patients had not been vaccinated. The age group 13–19 years was important with respect to the two doses of MMR vaccine required for school entry; however, no patients in this age group were identified in the IR system as having completed the two-dose MMR schedule.

**Table 1:** Demographic data and vaccination status for measles in Korea, 2013.

	Vaccination status				Total no. (%)
	0	1	2	Unknown	
**Sex**					
Male	30	16	2	6	54 (50.5)
Female	35	7	2	9	53 (49.5)
**Age group**					
1 year	46	0	0	0	46 (43.0)
0–5 months	5	0	0	0	5 (4.7)
6–11 months	41	0	0	0	41 (38.3)
1–3 years	12	7	0	0	19 (17.8)
4–6 years	2	1	2	0	5 (4.7)
7–12 years	1	0	1	0	2 (1.9)
13–19 years	1	13	0	1	15 (14.0)
≥20 years	3	2	1	14	20 (18.7)

We identified 4 imported cases and 103 import-related cases. The first imported case was reported in week 5 of 2013. The patient was a 24-year-old female of Thai nationality. Her rash occurred in February, and there was no further transmission. The second imported case patient, a 33-year-old male, was reported in early May; he became ill after a trip to Thailand in April. The third imported case involved a 7-year-old girl who had traveled to Indonesia in October, and the fourth imported case patient was a 2-year-old girl who became infected on a trip to the Philippines in November.

Of the four measles outbreaks in 2013, the initial cases were located in Gyeongnam, northern Gyeonggi, southern Gyeonggi, and Seoul (Fig 2). The southern Gyeonggi and Seoul outbreaks started with the third and fourth imported case, respectively.

**Fig 3:**
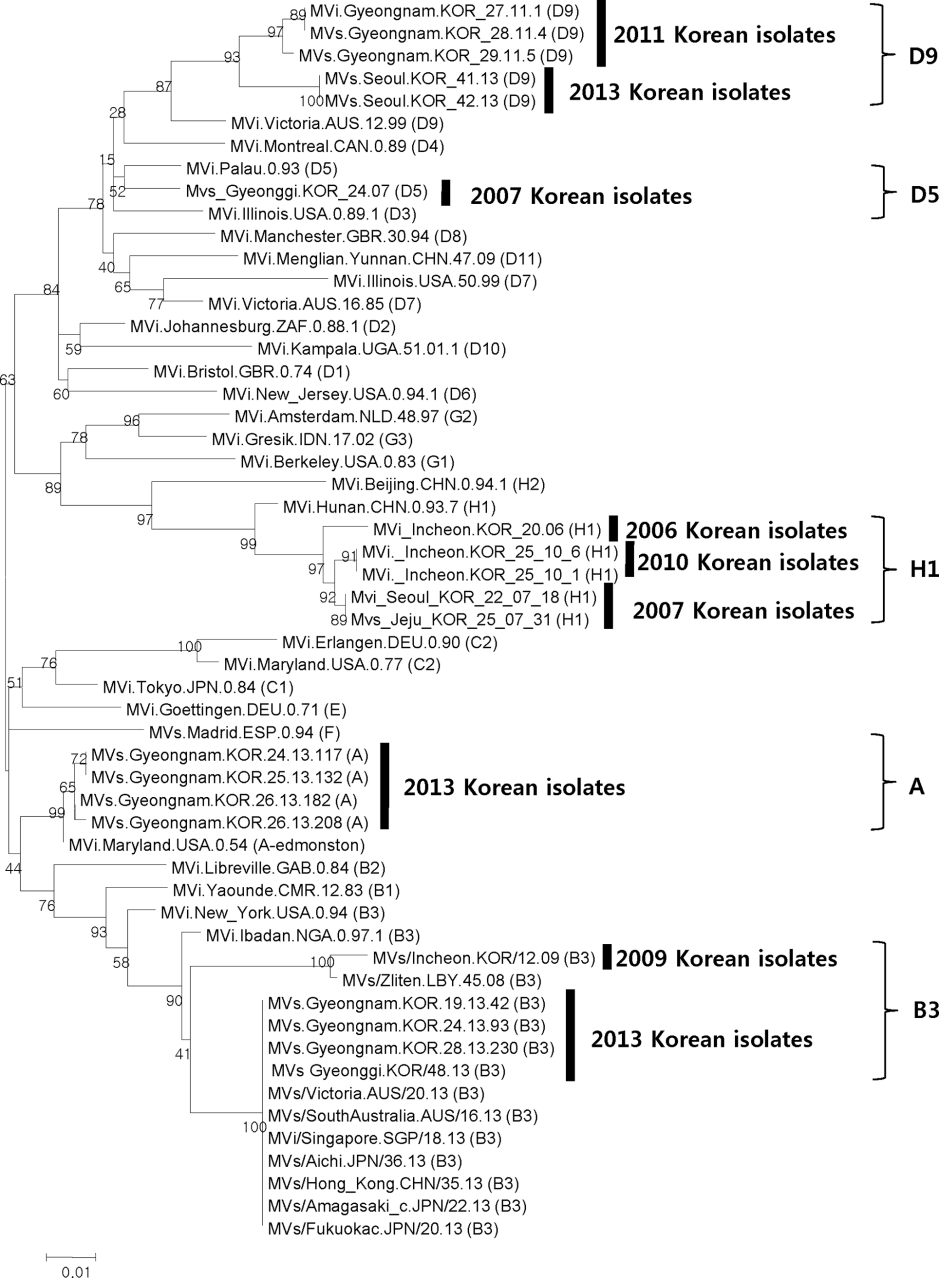
Phylogenetic tree of measles virus isolated in South Korea, 2006–2013.

In the 76 cases for which sequence analysis was performed, the B3 type (n = 74) and D9 type (n = 2) were confirmed (Table 2). In two of the sporadic cases, namely, the first and second imported case, the type of virus could not be identified; however, the circulating viruses confirmed that the B3 type was responsible for the three outbreaks in Gyeongnam and in northern and southern Gyeonggi whereas the D9 type circulated in Seoul. Although we could not identify the index cases in two of the outbreaks (Gyeongnam and northern Gyeonggi), the B3 type virus was circulating during both outbreaks. Measles virus isolation in South Korea during 2006–2013 revealed that H1 type was isolated in 2006, 2007, and 2010; D5 type was isolated in 2007 (Fig 3). The B3 type had not been in circulation during the previous 3 years in South Korea; this virological evidence suggests that these outbreaks were imported-related [14].

**Table 2:** Measles cases by source of infection in 2013 outbreaks.

	Total no.	Source of infection		Genotype (n, %)
		I	IR	
Total no. (%)	107	4 (3.7)	103 (96.3)	-
**Sporadic case**	2	2^†^	0	-
**Outbreak cases**				
Gyeongnam	74	0	74	B3 (53, 71.6)
Northern Gyeonggi	25	0	25	B3 (17, 68.0)
Southern Gyeonggi	4	1	3	B3 (4, 100.0)
Seoul	2	1	1	D9 (2, 100.0)

Abbreviations: I, imported; IR, import-related.

† These sporadic cases were identified as imported from Thailand, based on epidemiological evidence. Patients were located outside of Korea during the incubation period; genotyping failed owing to delayed medical notification, which prevented timely acquisition of specimens for sequencing.

#### Outbreak description

In 2013, 98.1% of cases (n = 105) were linked to one of outbreak the four outbreaks that occurred in Gyeongnam, northern and southern Gyeonggi, and Seoul. We also identified that measles occurred at one school, four hospitals, and four households (Table 3).

**Table 3:** Four measles outbreaks by region and vaccination status of case patients.

Region	Outbreak details				Vaccination status (%)			
	Size	Mean age (range)	Duration (weeks)	Transmission setting ^†^	0	1	2	Unknown
Gyeongnam	74	18 y (5 mo—40 y)	20	School (1), Hospital (4), Household (5)	39 (52.7)	21 (28.4)	4 (5.4)	10 (13.5)
Northern Gyeonggi	25	27 y (16 d—37 y)	13	Hospital (1), Household (4)	21 (84.0)	1 (4.0)	0 (0.0)	3 (12.0)
Southern Gyeonggi	4	1 y (11 mo—2 y)	2	Hospital (1)	4 (100.0)	0 (0.0)	0 (0.0)	0 (0.0)
Seoul	2	5 y (4–7 y)	3	Household (1)	1 (50.0)	1 (50.0)	0 (0.0)	0 (0.0)

† Location of additional measles cases during the outbreak.

The Gyeongnam outbreak began in early April 2013 at A high school, where 15 students were infected. One student at A school was treated in B hospital, and 1 additional case occurred at this hospital. The school outbreak ended in early May; however, 32 people were infected during the next 2 months in C hospital. Three patient from C hospital infected family members and 1 from C hospital was linked to D hospital cases. One community case patient, reported in June, was treated at E hospital, after which 7 additional children and their parents were infected. A further 2 household cases were linked to patients with measles in E hospital. During the Gyeongnam outbreak, a total of 10 patients that had acquired the disease in the community were not directly linked to other measles cases. Consequently, 44 case patients (59%) were exposed to measles during hospitalization or outpatient visits to a medical institution.

**Fig 4:**
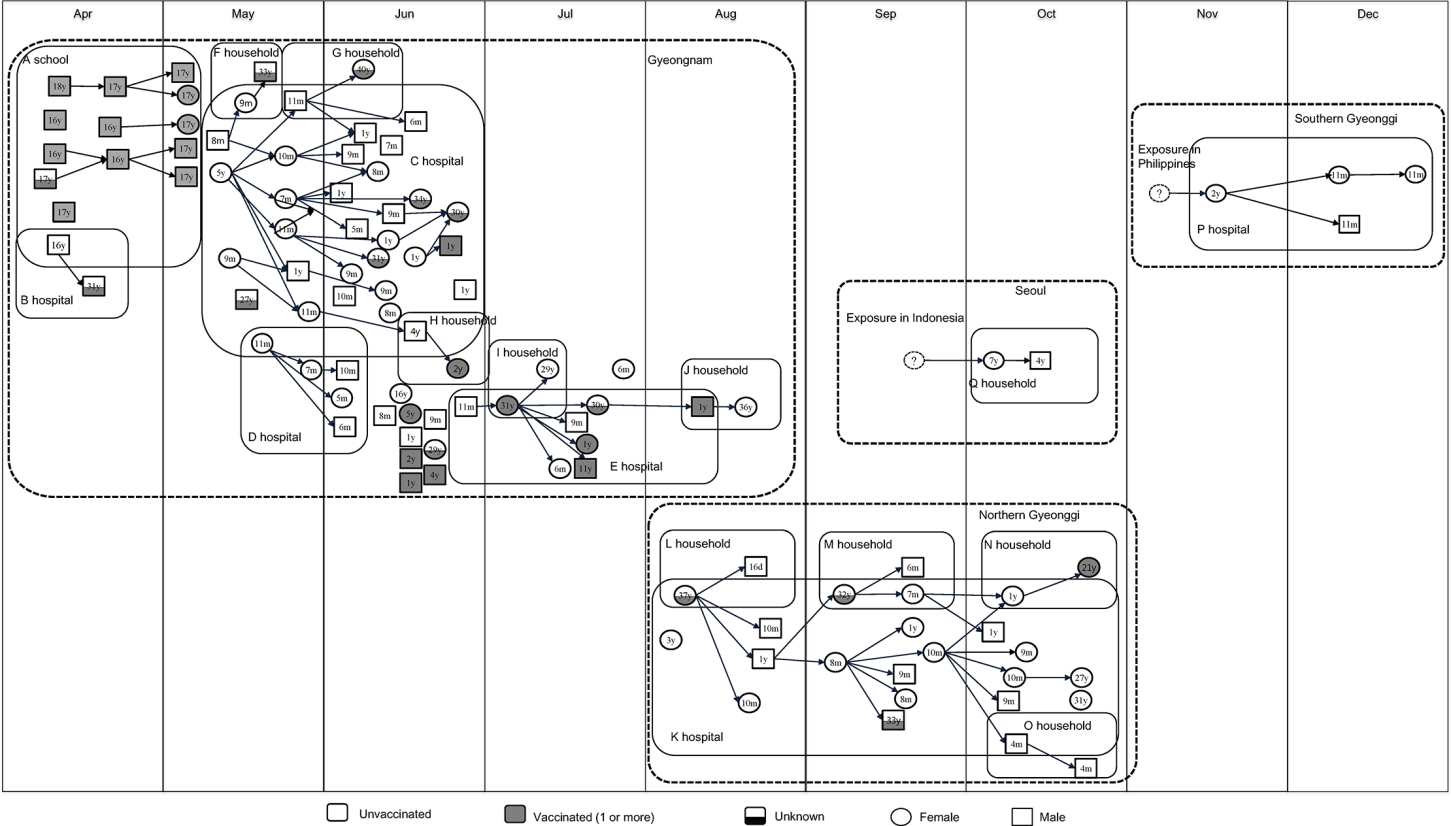
Transmission patterns of measles outbreaks in four regions of South Korea, 2013.

In the Gyeongnam outbreak, the median age of patients was 18 years (5 months to 40 years), and 53% of cases involved children with no immunization history (Table 3). The mean generation time was 14 ± 4.0 days. The average generation time in A school (9 days) was shorter than the total average. Schools provide an environment in which students spend all day in the same crowded space. Generation time in the households was 12 days; however, in G household where family members did not live together, the generation time was 19 days longer than the overall average (Fig 4).

A total of 25 cases were reported in the northern Gyeonggi outbreak, which lasted for 13 weeks from August to October, 2013 (Figs 1 and 2). The index case was a 37-year-old female patient. She was infected with measles during pregnancy and was treated in K hospital during the infectious period. Four additional cases involved infants and young children who visited K hospital and became infected with the disease. A neonate who was born to the index case developed a typical rash at 16 days after birth; laboratory tests were positive for IgM and RT-PCR but not IgG (titer: 244).

The northern Gyeonggi outbreak primarily involved unvaccinated children (68%). The median age of case patients in this outbreak was 27 years (16 days to 37 years); the mean generation time was 14 ± 2.7 days.

The southern Gyeonggi outbreak involved 4 cases and lasted for 4 weeks from November to December 2013. The index case involved a 2-year-old child who became infected with measles after traveling to the Philippines and then visited P hospital when he returned to South Korea. Three additional cases were reported in southern Gyeonggi. All four cases involved unvaccinated children, and three involved infants. The median age was 1 year (11 months to 2 years) and the mean generation time was 15 ± 2.7 days.

The last measles outbreak was in Seoul, which involved two cases. The index case patient was a 7-year-old girl who was exposed to measles in Indonesia in mid-September 2013. Her younger brother, who was 4 years old, was infected by the index case. Neither had received any vaccination. The median age was 5 years (4–7 years), and the generation time was 11 days.

## Discussion

In 2013, measles primarily affected infants (43.0%); this high incidence among infants may be related to the duration of the maintenance of passive immunity from the mother. The duration of protective passive immunity differs and depends on the amount of antibodies transmitted from the mother [16]. The amount of antibodies received from a mother with vaccine-induced immunity is lower than that transmitted from a naturally immunized mother [17]. In recent times, the mother is more likely to have acquired measles immunity through vaccination. In South Korea, the recommendation is for two doses of MMR vaccine, with the first dose given at age 12–15 months, and the second dose at age 4–6 years. Furthermore, there have been no large measles outbreaks since 2001 and South Korea has maintained high vaccination coverage of two doses of MMR vaccine (more than 95%) [18, 19]. A seroprevalence study in 2010 showed that the positivity rate for measles antibodies in infants was 70%, but this rate decreased rapidly after the age of 5 months [20]. Additionally, we found that 82.6% of infants were infected when they were admitted to hospitals or to an outpatient department (Fig 2). It is more likely that complications occur after mild illness in younger patients than in older ones, and young patients require more medical care. Therefore, there are more opportunities for exposure to infectious diseases at a medical facility among younger children, who are thus more susceptible to measles infection.

In the prevaccine era, measles could affect an entire birth cohort. However, since the elimination of measles in the US, in 2000, the ages of people affected by measles has depended on the outbreak setting. From 2001 through 2008 in the US, the age group most affected by measles was infants <1 year. In 2002, most cases occurred in infants because of an outbreak at a childcare center. In 2006, an outbreak occurred in a large office building and most case patients were aged 20–39 years.

In this study, the proportion of adolescent measles cases was 14% (n = 15). Two students at A high school in the early stage of the Gyeongnam outbreak were freely active for a total of 16 days after disease onset, as their diagnosis was delayed by a week. Delayed recognition of measles infection in students at A school led to continuous exposure of other adolescents to the measles virus. Delayed recognition was caused by the atypical measles symptoms of mild fever and rash among students. In 2010, students infected with measles at a middle school in the city of Incheon showed an atypical clinical course of measles [8]. Those students may have had a less than optimal measles immunity status, and they were continuously exposed to measles at school. Future studies should assess the decreased duration of passive immunity in infants and adolescents and evaluate the long-term effect of this on maintaining herd immunity.

In the present study, we found that 61% of case patients became infected at medical institutions. A hospital provides many opportunities for contact between infected and susceptible people, and South Korea has easy access to medical institutions [21]. Persistence of measles in hospitals indicates that the current approaches to infection control and prevention are insufficient. Measles is a contagious disease even before a rash appears. Control measures should take priority if a child develops a fever and respiratory symptoms, and particularly if a primary measles case patient has recently visited a doctor’s office or hospital ward during the infectious period.

To interrupt and mitigate measles transmission in the four provinces discussed here, local governments have emphasized the importance of reporting suspected measles cases and immediate isolation of individuals who develop measles-like symptoms or illness. In addition, public health centers, hospitals, child care centers, and schools should be monitored (i.e., daily zero reporting). Outbreak information should also be distributed to clinicians and the public via the press, mass media, and bulletins to increase measles awareness.

In response to the outbreak at A school in Gyeongnam Province, the KCDC initiated active surveillance in schools and provided supplementary immunization with one dose of MMR vaccine for 782 students, 68 school staff and teachers, and 5 family members who had no record in the IR system of having received a second dose of MMR vaccine.

Measles cases in 2013 were caused by patients or viruses entering South Korea from outside the country [22]. The epidemiological evidence showed that some patients were clearly infected during an overseas trip, and most cases were associated with outbreaks that were import-related epidemiologically. Even when the origins of an outbreak were unknown based on the epidemiological evidence, virological evidence of the virus type supported that the outbreak was import-related. The B3 type was the predominant circulating virus in South Korea in 2013. Before 2013, the B3 type had only been detected in one imported case, a patient of Libyan nationality, in 2009 [7, 10, 11,12].

The four outbreaks in Korea terminated in 2013, and the KCDC declared the end of all outbreaks after confirming that no further cases had been identified 42 days from the onset of the last case.

A limitation of this epidemiological study was that the index case in two outbreaks was unknown, as was the transmission route for the community-acquired case in the Gyeongnam outbreak. This may indicate a shortcoming in the surveillance system. Despite this limitation, we found evidence that all outbreaks originated from imported or import-related viruses, based on molecular analyses. We were able to follow the patterns of transmission of all outbreaks during the year, using available national surveillance data. This study is important for the implementation of strategies to sustain measles elimination in Korea.

## Conclusion

Epidemiological and molecular evidence strongly supports that the sources of measles in South Korea in 2013 came from outside the country. Although Korea has maintained a high level of immunity for the entire population of the country, an environment in which susceptible people were temporarily in close contact with infected ones may have caused the 2013 measles outbreaks [23]. Similar to the US and Australia, countries where endemic measles virus has been eliminated, there is a need to sustain the elimination status of measles in Korea, as the virus continues to be imported into countries that have been declared free of measles [24]. Importation of measles can occur at any time, but especially during the holiday season; there is also a potential risk of nosocomial transmission after international importation of measles. This highlights the need for people to be immunized before traveling and for awareness among healthcare workers. It is also necessary to provide information (endemic countries, prophylaxis methods, measles symptoms, and so on) to individuals, including physicians, for early detection of the disease. Furthermore, timely application of comprehensive infection prevention and control measures are imperative.
